# SARS-CoV-2 Emergency and Long-Term Cognitive Impairment in Older People

**DOI:** 10.14336/AD.2021.0109

**Published:** 2021-04-01

**Authors:** Maria Rosaria Rizzo, Giuseppe Paolisso

**Affiliations:** Department of Advanced Medical and Surgical Sciences, University of Campania “Luigi Vanvitelli” Piazza Miraglia, Naples, Italy

**Keywords:** SARS-CoV-2 infection, Long-term cognition, SARS-CoV-2 neurotropism, Older people, ACE2, Cognitive assessment

## Abstract

The SARS-CoV-2 infection has spread to all continents, affecting particularly older people. The complexity of SARS-CoV2 infection is still under study. Despite respiratory involvement is the main clinical manifestation of COVID-19, neurological manifestations are common. Although it is obvious to give priority to infectious emergency and the infectious disease itself, at present, however, data on potential long-term damages generally and on long-term cognitive functions impairment of older COVID-19 survivors have yet to be investigated. Because the hypothesis on the involvement of SARS-CoV-2 on the long-term cognitive decline pathogenesis would seem difficult to prove, we wanted to explore the brain mechanisms of SARS-CoV-2, in order to provide more in-depth analysis and to draw attention to a topic relevant to basic scientific research and, more generally, to the elderly population.Looking forward, we argue that an early clinical and instrumental cognitive assessment can help prevent and slow down this possible complication or at least improve the quality of life for older people Covid-19 survivor.

A large amount of knowledge about Coronavirus disease (COVID-19) pandemic is available, but much less is known about what could happen after surviving this disease. Although the recent pandemic has spread to all continents, affecting millions of people, and unfortunately it is not yet in a phase of "fading", we are still trying to understand the complexity of severe acute respiratory syndrome (SARS) by Coronavirus 2 (SARS-CoV-2 infection) and its sequelae. All age groups have been and are at risk of contracting COVID-19, but older people are at higher risk of developing severe illness due to physiological changes linked to the aging process in itself and to comorbidities presence.

Reports show that over 90% of deaths occurred in older people and more than 50% of them aged 80 years and above [WHO. World Health Organization - Situation Reports (2020). Available from: www.who.int/docs/default-source/coronaviruse/situation-reports/20200315-sitrep-55-covid-19.pdf?sfvrsn=33daa5cb_6]. Moreover, we can confirm that this infection affected people 65 years and older living in nursing homes or long-term care [1]. Therefore, Covid-19 has highlighted the vulnerability of older people to acute diseases. In most cases, the main clinical manifestations of this disease are the same as the common cold (fever, fatigue, sore throat, cough), but can rapidly progress to mild to very severe respiratory symptoms up to full-blown respiratory failure, associated with all the consequences of prolonged hypoxia [2]. When hospitalization occurred in an Intensive Care Unit (ICU), this had other significant health consequences such as failure or dysfunction of multiple organs, like kidneys, heart, and brain [3].

Other symptoms were also observed, such as gastrointestinal involvement (nausea and diarrhea), and cardiovascular damage [4], but what we believe to be of extreme importance for older people is the report of neurological symptoms.

In fact, almost 30% of the population affected by COVID-19 experienced headaches or dizziness, epileptic crises, neuralgia, and in many cases Guillain-Barré syndrome, muscle weakness, severe encephalitis or altered consciousness, stroke, and loss of smell and taste [5]. These last symptoms, suggesting a convincing neurotropism, often appeared before the pulmonary ones but were considered less significant than the most shocking respiratory manifestation.

Already Bergman et al [6] by analogy with the neurotropism of other coronaviruses, mainly SARS-CoV-1, MERS-CoV, and OC43, suggested brain invasion also for SARS-CoV-2. In particular, the neurological invasion or so-called neurotropism has been demonstrated in all three epidemics caused by coronaviruses [7-8].

In a recent review, Almqvist et al [9] underlined that SARS -CoV-2 appears to have the same role as SARS-CoV-1 in the nervous system. The authors focused also on the similarities in neurological symptomatology across SARS-CoV-1 and SARS-CoV-2, highlighting the need for neurological complications monitoring in patients infected by SARS-CoV-2, because neurological complications may result in chronic disability. Even in studies made in animal models, the authors demonstrated that the brain was for SARS-CoV2 a target organ. [10].

Anyway, there are many studies and reviews focusing on the neurological manifestations related to coronavirus infection in humans, but none of these studies consistently addressed any cognitive impairment assessment. Therefore, in our opinion, in addition to researching the mechanisms that make the brain a target for SARS-CoV-2, it is of great importance to continue investigating how SARS-CoV-2 enters the central nervous system so infecting the neuron cell. Even more, given possible neurotropism [11], it is urgent to understand whether the SARS-CoV-2 infection may be able of developing a worsening of cognitive functions, particularly in elderly patients. Several aspects of COVID-19 are likely to impact on cognition.

Here, we provide a summary of the brain involvement in COVID-19, focusing, in particular, on SARS-CoV2 neurotropism mechanisms, brain expression receptors, hypothalamic tropism as well as on the inflammation and hypercoagulable state.

Finally, we will hypothesize how post- SARS-CoV2 infection nervous damage could result in cognitive impairment. Moreover, in order to minimize cognitive impairment probably due to SARS-CoV2, we will try to recommend a path to facilitate the diagnostic work designing a specific screening for these complications.

**Figure 1.**
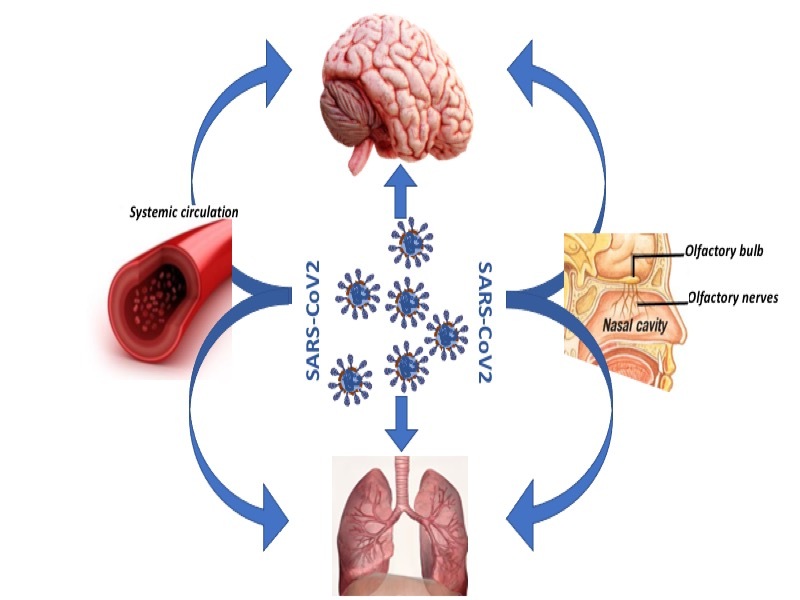
SARS-CoV2 main pathways to reach the brain: olfactory nerves and/or diffusion into the systemic circulation.

## How does the SARS-CoV-2 virus affect the brain?

Many authors have hypothesized that the virus gets to the brain essentially through two main pathways: through the cribriform plate of the ethmoid bone and up to the olfactory bulb and from there to the olfactory cortex and/or through the diffusion of the systemic circulation [12-13] [Fig.1].

A potential mechanism of entry into the brain of SARS-CoV-2 is the use of the axonal transport mechanism, infecting peripheral neurons [12,14]. In fact, SARS-CoV-2, from the nose and through the upper respiratory passages, reaches the brain, through the olfactory nerve that projects the dendrites into the nasal cavity and through the cribriform plate that projects the axons into the olfactory bulb. This explains hyposmia and hypogeusia as neurological manifestations reported in some cases [12,14].

Other mechanism is the dissemination of SARS-CoV-2 through the hematogenous pathway, directly infecting the endothelial cells of the BBB, thus made more permeable, or by infecting the leukocytes themselves, used by the virus as a transporter to migrate through the blood-brain barrier (BBB) in the brain [13-14] or by infecting leukocytes and migrating through the BBB [13-14].

The tropism of SARS-CoV-2 is therefore dependent on the expression of cellular proteases, as well as activating Angiotensin-Converting Enzyme 2 (ACE2). SARS-CoV-2 can cause neurological disorders as a result of entry inside neuronal cells through ACE2 protein receptor [12-15]. In particular, ACE2 is a transmembrane protein characterized for its role in counterbalancing the effects of ACE on the cardiovascular system. ACE2 is expressed in both neurons and glia, thus allowing the virus RNA replication and alter and/or kill the neuronal cell [12-15]. Thus, SARS-CoV-2 binds to the ACE2 in order to enter in target cells. These receptors are abundant in the cells of many human tissues, including the brain. Molecular and experimental studies have shown that SARS-CoV-2, as well as SARS-CoV, with the S1/S2 subunit of the glycoproteins of its spikes, is able to bind to the ACE2 receptor. The subsequent endocytosis of ACE2 linked to SARS-CoV2, allows the virus to enter the cell, thus causing a reduction of the ACE2 receptor expressed on the cell surface. Overall, the downregulation of ACE2, observed in animal models infected with SARS-CoV and SARS-CoV-2, causes an imbalance in ACE/ACE2 activity with a consequent increase in tissue damage [12-15].

SARS-CoV neurotropism is confirmed also in animal models. In fact, in mice transgenic for human ACE2 (hACE2 mice) the brain was the principal target organ for SARS-CoV [10-16]. Nevertheless, the majority of these studies have focused on the brain generally. In effect, the hypothalamus region is rich in ACE2, at least in animals [16]. However, also in post-mortem human brain tissue, ACE2 has been observed in the hypothalamus region [17-19]. Previously, some researchers have analyzed the presence of ACE2 in a number of hypothalamic nuclei (insula, amygdala, myelencephalon and pons) and connected regions noting that gene expression levels varied widely between the brain regions studied [20]. Intriguingly, ACE2 expression levels were highest in the paraventricular nucleus of the hypothalamus and choroid plexus, in agreement to its role towards the renin-angiotensin system [17-19]. From here, the evidence that the hypothalamus areas are the main potential targets of SARS-CoV-2 [17-19].

Li et al demonstrated that in addition to direct lung injury, SARS-CoV-2 could also affect the cardiorespiratory system indirectly via the medulla oblongata (brainstem) [11].

Whether pulmonary and extrapulmonary ACE2 expression is the best-known mechanism of SARS-CoV-2 infection, at the same time, not-ACE2 pathways should not be excluded.

There is no doubt several other networks that are involved in SARS-CoV-2 pathogenicity. In addition to damage from direct brain infection, SARS-CoV-2 can cause indirectly neurological disorders as a result of the cytokines storm, elevated D-dimer levels and thrombocytopenia [21-22], making patients prone to cerebrovascular events, both thrombotic and hemorrhagic [21-22].

Most COVID-19 patients exhibit increased circulating levels of IL-6, IL-1b, and TNF, as well as IL-2, IL-8, reflecting the disease severity, which results in massive systemic immunosuppression [23-24] and in lymphopenia and neutrophilia, two key hematological features of COVID-19 [25-26].

After brain entry, these molecules induce an innate immune response, particularly in pericytes, macrophages, and microglia, increasing even more cytokine production and impairs brain function [27].This immune dysregulation could contribute to the alterations in consciousness. Moreover, intracranial cytokine storms, which could result in the breakdown of the BBB, without direct viral invasion, might be responsible for the development of acute encephalopathy or Guillain-Barré syndrome [28].

Another feature of COVID-19 is a hypercoagulable state [29-30] characterized by a pro-coagulant state, increased D-dimers and increased fibrinogen, which may start in the lungs and also in other infected organs. In fact, SARS-CoV-2 may promote a dysregulated platelet activity also directly through the angiotensin receptors expressed on the surface of platelets, or indirectly by the coagulation cascade regulation [29].

**Figure 2.**
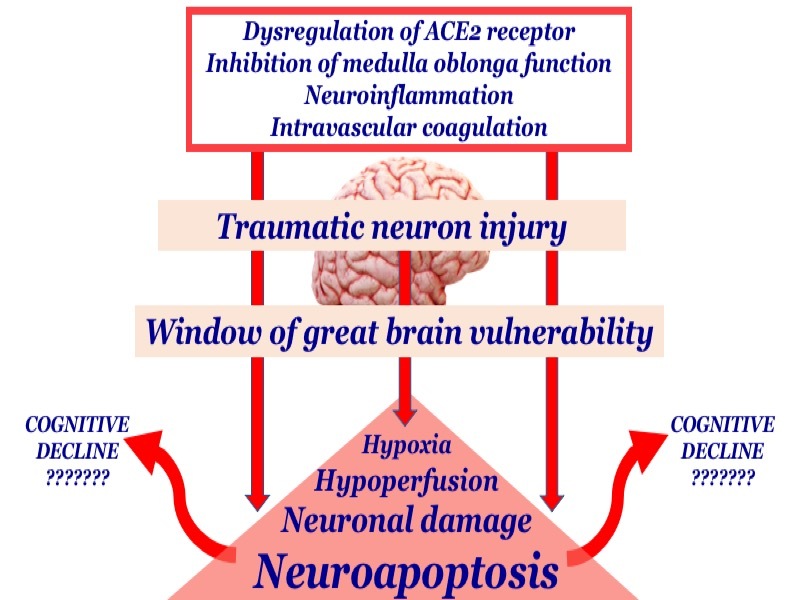
Potential brain mechanisms contributing to increased risk of cognitive decline during and after COVID-19.

In addition, also considering the mode of occurrence of SARS-CoV2, characterized by violence, rapidity and sudden spread in the population, we can hypothesize that SARS-CoV-2 can easily have neuroinvasive capacity because it creates a "window of greater brain vulnerability". This scenario together with hypoperfusion, hypoxia, neuroinflammation, and blood-brain barrier dysfunction could, in turn, amplify neuronal damage and apoptosis [31] [Fig. 2].

Consequently, the exact pathway in the nervous system could be just any of these routes or their combination, but in our opinion, cannot exclude that brain damage from acute SARS-CoV2 infection can be comparable to severe traumatic neuron injury. Due to these interesting possible propagation mechanisms, once entered the brain, SAR-CoV-2 could cause damage to multiple brain areas, such as the cortex and hypothalamus.

As known the hypothalamus, together with the amygdala, the reticular formation of the brain stem, and other portions of the brain, takes part in the constitution of the limbic system, which plays a key role in emotional reactions, memory processes, emotions, attention, learning, behavior, and smell [32].

### Do people who survived to COVID-19 pandemic have to worry about their long-term cognitive performance?

Despite lung function recovery, Covid-19 survivors could experience a high prevalence of impairments including increased risk for mental health issues like persistent psychological disabilities, post-traumatic stress disorder (PTSD) [33], anxiety, and depression but they may also have a significant reduction of physical ability [34-35] as well as a worsening in cognitive performance.

It is known that age is a risk factor for the manifestation of main disease complications, as well as neurologic component worsening and cognitive decline. Therefore, we urge all to remember that older people are already at the highest risk of cognitive decline and dementia.

Yet, neurological and cognitive symptoms due to SARS-CoV-2 constitute a clinical scenario different from the classic and better-known respiratory manifestation, but even more, worrying for the elderly population that survived to COVID-19. In other words, one should be aware that classical neurological manifestations due to COVID-19, both during and post-acute illness could also be an indicator of a possible long-term cognitive worsening.

Unfortunately, there is insufficient data to know the extent of the consequences of infection on long-term cognitive performance, nor information on how to distinguish classic age-related cognitive impairment from that actually caused by SARS-CoV-2. In fact, it's really difficult to say what's going to happen without evidence.

In any case, it is our interest precisely with this paper to stimulate the attention of researchers and the general population on a topic that we believe is of strong importance.

Actually, there are very few studies reporting cognitive dysfunction related to COVID-19. Most of the studies address the presence of neuropsychological disorders [36], and among these only one study shows that SARS-CoV-2 infection is associated with lower cognitive function [36]. Another study showed that 6 cases of 125 COVID-19 hospitalized patients with neurological manifestations presented a neurocognitive disorder [37]. Another one showed that some COVID-19 hospitalized patients had lower cognitive scores on a telephone interview after 4 weeks of discharge [38]. In a retrospective study of 50 hospitalized patients with COVID-19 who presented neurological symptoms, 24% of them had short-term memory loss [39].

As evident, the data on cognitive performance are few, probably because the respiratory symptoms have been much more striking and demanding to deal with, while the neurocognitive symptoms have often gone unnoticed as if it were a "niche phenomenon" such as not to require either attention and even less the classic complete neuropsychological evaluation.

Therefore, we deliberately addressed the problem of cognitive performance in elderly patients who survived COVID19, clearly expressing the poverty of current knowledge, today still hovering between hypotheses and a few pieces of evidence.

However, the causality and mechanisms involved in such association remain to be elucidated and accepted as a mechanism of neurological injury. Worthy of attention is the fact that a large number of studies have indicated that exposure to various microbial pathogens, viruses, in particular, can accelerate Dementia pathology, suggesting that certain types of infections may play a significant role in Alzheimer's pathogenesis. This is important data because if viral infections increase the risk of AD, the viruses themselves represent a modifiable risk factor that can be treated with the therapeutic intervention [40].

Furthermore, we cannot either rule out the possibility that SARS-CoV-2 may remain latent in the central nervous system. Added to this, due to the time of isolation and quarantine, levels of loneliness, depression, and worsening behavior are also expected to rise. Consequently, in our opinion, just now we should step up the investigation of brain damage in any area, by using clinical, serological, and radiological methods. The individuation of a neuronal biomarker and the use of the radiomics, both valid tools to identify the extent and the effects of brain damage, would be of enormous help to provide insights into the degree of cognitive decline in order to improve the quality of elderly survivors [41]. As a result, the evidence-based information obtained by CT and/or MRI and related to neurological function and manifestations must be taken into consideration immediately to show that SARS-CoV-2 infection is truly related to dementia.

Based on our clinical experience, we believe that we will be able to have an adequate follow-up of cognitive function, only if the radiological approach to the brain is practiced early and already in this phase of the pandemic. Using radiomics analysis, all the features extracted from multiparametric magnetic resonance imaging (MRI) can be used as potential biomarkers of preclinical dementia, so offering an opportunity for early intervention [42].

This idea highlights also the need for longitudinal studies in aged people in order to examine the impact of COVID-19 and its effect on cognitive performance. If we can delineate new diagnostic strategies of effective cognitive assessment in the elderly, during, and after era COVID-19, we might also be able to develop better cognitive assessment in the general population.

### Why is it so important to screen the elderly patient’s cognitive performance?

The answer to this question is critical to the future of global health. Very often the cognitive function assessment in routine clinical practice is undervalued, although various tools are available [43]. In most cases, cognitive decline is self- reported as worsening or confusion or loss of memory, while other times the family members are the ones to report the patient's cognitive impairment. Cognitive decline can have implications on basal and instrumental activity daily living, thus reducing the degree of autonomy of the elderly patient and increasing the burden on the caregiver. In its heterogeneous manifestation, mild cognitive impairment could be a stable situation or the earliest symptom of more serious and degenerative dementia [44].

Hence, at this particular moment, to focus on the possible cognitive impact of the SARS-CoV-2 infection is almost an obligation. All patients could develop worsening of cognitive function after COVID-19, especially those who survived the Intensive Care Unit (ICU). In this regard, we are already trying to program and develop new strategies for cognitive decline prevention and control in older COVID-19 survivors.

It will not be easy to differentiate to what extent the cognitive decline can be attributed directly to SARS-CoV-2 infection as opposed to encephalopathy due to the hypoxia acute, or to the systemic neuro-inflammation due to the infection response, or also to the effects of the therapies used. However, improving the understanding of the possible cognitive decline cause could lead to effective treatments, which are vital to tackle this already increasingly serious public health problem. Also, we believe the "COVID-19 cognitive impact" should be analyzed not only in terms of mechanisms of cell damage but also in terms of brain repair processes. As an example, neurotrophic factors are known to play an important role in neurogenesis and in neuronal repair and this led to the development of effective strategies on post-stroke therapy and in the potential treatment of dementia and brain injury [44-45]. In particular, treatment with the neurotrophic protein S100B, which is a marker of brain injury, following experimental brain injury increases hippocampal neurogenesis in rodents, improving their cognitive function [45-46].

### Conclusion and future directions

In public mental health terms, the SARS-CoV-2 impact on cognitive performance could represent now a major concern, along with mental health and physical well-being. Thus, our hypothesis in the first-place invites interested researchers to investigate the link between SARS-CoV-2 and possibly long-term cognitive decline, but it could also motivate additional actions useful to improve the health and well-being of older people who survived COVID-19. Because delay of diagnosis can be the main cause of cognitive decline, timeliness is critical to reducing the number of dementia new cases. In presence of evidence, public health decision-makers could be pushed to consider the long-term decline in those patients who survived the COVID-19 as a priority; health care professionals could increase their awareness of cognitive decline assessment and management; the same patients could be encouraged to contact the specialist for an early cognitive assessment.

In the end, we suggest tight monitoring of high-risk elderly patients, using applicable cognitive assessment clinical and radiological methods in addition to appropriate medications when needed so that long-term effects of the complications of this pandemic on the cognitive function and disability can be the subject of clinical and basic research in the immediate future.
